# Development and Evaluation of a High-Throughput Single-Nucleotide Polymorphism Array for Large Yellow Croaker (*Larimichthys crocea*)

**DOI:** 10.3389/fgene.2020.571751

**Published:** 2020-10-23

**Authors:** Tao Zhou, Baohua Chen, Qiaozhen Ke, Ji Zhao, Fei Pu, Yidi Wu, Lin Chen, Zhixiong Zhou, Yulin Bai, Ying Pan, Jie Gong, Weiqiang Zheng, Peng Xu

**Affiliations:** ^1^Fujian Key Laboratory of Genetics and Breeding of Marine Organisms, College of Ocean and Earth Sciences, Xiamen University, Xiamen, China; ^2^State Key Laboratory of Large Yellow Croaker Breeding, Ningde Fufa Fisheries Company Limited, Ningde, China

**Keywords:** NingXin-I, SNP, array, genotyping, large yellow croaker, Sciaenidae

## Abstract

High-density single-nucleotide polymorphism (SNP) genotyping array is an essential tool for genetic analyses of animals and plants. Large yellow croaker (*Larimichthys crocea*) is one of the most commercially important marine fish species in China. Although plenty of SNPs have been identified in large yellow croaker, no high-throughput genotyping array is available. In this study, a high-throughput SNP array named NingXin-I with 600K SNPs was developed and evaluated. A set of 82 large yellow croakers were collected from different locations of China and re-sequenced. A total of 9.34M SNPs were identified by mapping sequence reads to the large yellow croaker reference genome. About 1.98M candidate SNPs were selected for further analyses by using criteria such as SNP quality score and conversion performance in the final array. Finally, 579.5K SNPs evenly distributed across the large yellow croaker genome with an average spacing of 1.19 kb were proceeded to array production. The performance of NingXin-I array was evaluated in 96 large yellow croaker individuals from five populations, and 83.38% SNPs on the array were polymorphic sites. A further test of the NingXin-I array in five closely related species in Sciaenidae identified 26.68–56.23% polymorphic SNP rate across species. A phylogenetic tree inferred by using the genotype data generated by NingXin-I confirmed the phylogenetic distance of the species in Sciaenidae. The performance of NingXin-I in large yellow croaker and the other species in Sciaenidae suggested high accuracy and broad application. The NingXin-I array should be valuable for quantitative genetic studies, such as genome-wide association studies (GWASs), high-density linkage map construction, haplotype analysis, and genome-based selection.

## Introduction

Large yellow croaker (*Larimichthys crocea*) is a commercially important marine fish species native to the northwestern Pacific, generally found in temperate waters. It is one of the traditional species favored by Chinese consumers. The large yellow croaker industry in China collapsed in the 1970s due to overfishing and recovered gradually since the establishment of artificial propagation in 1985 (Liu and De Mitcheson, 2008; Chen et al., 2020). The global production of large yellow croaker was 269,300 tons in 2016 [Food and Agriculture Organization (FAO)]. To date, more than 99% of the large yellow croaker was produced by China. Large yellow croaker is the top marine aquaculture species in China according to the annual yield (Bureau of Fisheries of the Ministry of Agriculture 2019). Recently, the large yellow croaker industry has encountered great challenges, including devastating diseases and environmental stresses, which caused a large amount of economic loss and hampered the healthy and sustainable development of the large yellow croaker industry (Bai et al., 2020). Large yellow croaker strains with a high level of disease resistance, fast growth rate, and high tolerance to stresses are desperately needed.

Because of the economic and biological importance of large yellow croaker, a lot of genomic and genetic resources have been developed. Lots of single-nucleotide polymorphism (SNP) markers were identified in large yellow croaker since the application of high-throughput sequencing technology (Xiao et al., 2015, 2016). Two linkage maps were constructed based on the identified SNP markers (Ao et al., 2015a; Kong et al., 2019). The draft genomes (Wu et al., 2014; Ao et al., 2015b; Mu et al., 2018) and chromosome level reference genome (Chen et al., 2019) of large yellow croaker were reported. The SNP markers, linkage maps, and genome sequences have also been used in genome-wide association studies (GWASs) to identify quantitative trait loci (QTL) associated with growth (Zhou et al., 2019) and body shape (Dong et al., 2019) of large yellow croaker.

Dissection of the genetic basis of these traits requires a large number of tools and resources such as genetic markers and high-throughput genotyping platforms. A major challenge for genetic research in large yellow croakers is the lack of a high-throughput genotyping SNP array. High-density SNP array and direct genotyping by sequencing (GBS) techniques, in particular, restriction site-associated DNA sequencing (RAD−Seq), are popular tools for population-level SNP genotyping (Li and Wang, 2017). Compared with RAD sequencing, the high-throughput SNP arrays showed higher repeatability and reproducibility, and more straightforward experimental procedures and bioinformatic analyses (Robledo et al., 2018). SNP arrays are efficient and robust tools for genome-scale genotyping, which have been developed in many fish species including the 250K common carp array (Xu et al., 2014); the 250K and 690K catfish arrays (Liu et al., 2014; Zeng et al., 2017); the 50K and 58K Nile tilapia arrays (Joshi et al., 2018; Yáñez et al., 2020); the 50K and 57K rainbow trout arrays (Palti et al., 2015; Salem et al., 2018); the 15K, 286K, and 400K Atlantic salmon arrays (Gidskehaug et al., 2010; Houston et al., 2014; Yáñez et al., 2016); the 50K Japanese flounder array (Zhou et al., 2020); the 6K giant tiger shrimp array (Baranski et al., 2014); the 9K Pacific white shrimp array (Jones et al., 2017); and the 38K and 190K oyster arrays (Gutierrez et al., 2017; Qi et al., 2017). The applications of the high-density SNP array in GWAS have identified QTL associated with various traits in multiple species, including growth, disease resistance, heat stress, hypoxia tolerance, morphometric, sex, and body conformation (Abdelrahman et al., 2017; Zenger et al., 2019). While genome-wide genotyping of large yellow croaker has been conducted using RAD-Seq (Dong et al., 2019; Wan et al., 2019; Zhou et al., 2019), no SNP array is currently available. Development of a high-density SNP array would accelerate GWAS and help to uncover the genetic basis of various traits in large yellow croaker.

Artificial fertilization and selective breeding programs of large yellow croaker have been established based in family selection (Yu et al., 2017). Marker−assisted selection (MAS) and genomic selection (GS) in fish breeding schemes would be especially valuable for traits that are difficult to record, such as disease resistance, filet quality, feed efficiency, and sexual maturation (Sonesson and Meuwissen, 2009). GS holds advantages over pedigree selection in breeding value prediction accuracy and the correspondence to genetic gain in breeding programs. The application of MAS and GS in breeding programs of aquaculture species is expected to be a fertile area of research (Yue, 2014). GS was successful in breeding for resistance against *Cryptocaryon irritans* in large yellow croaker (Zhao et al., 2021). With the availability of abundant genomic and genetic resources in large yellow croaker, in particular, SNPs, reference genome sequences, and high-throughput genotyping SNP array, the large yellow croaker breeding and selection would be accelerated. We report here the development and performance evaluation of the NingXin-I SNP array using the Affymetrix Axion genotyping technology.

## Materials and Methods

### Sample Collection and Genome Re-sequencing

**FIGURE 1:**
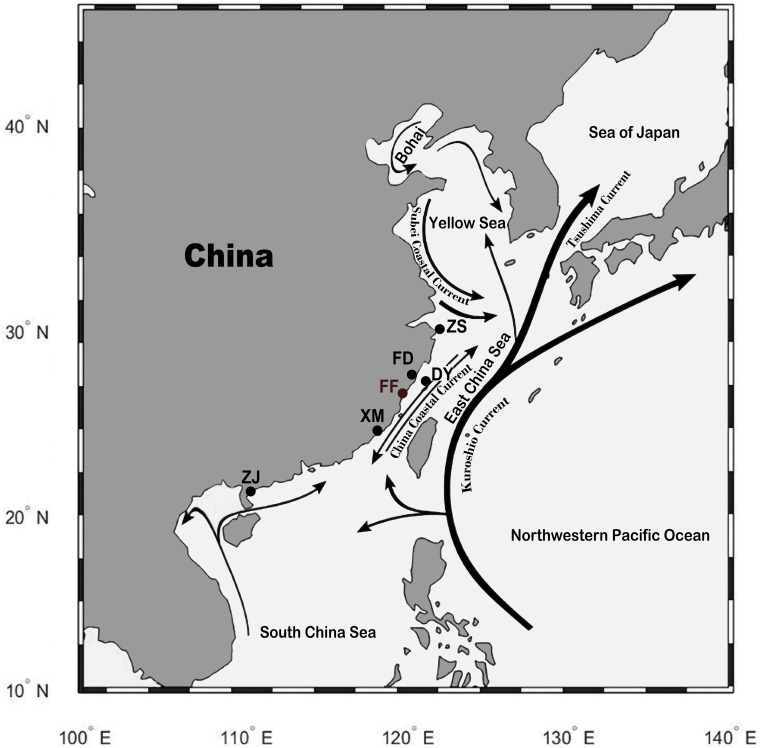
Sampling location of the wild and cultured population of large yellow croakers. Wild population: ZS, Zhoushan; FD, Fuding; DY, Dayu; XM, Xiamen; ZJ, Zhanjiang. Cultured population: FF, Fufa. The black lines with an arrow indicate the current in the sea. The figure was generated by using the R package “ggmap” (author David Kahle).

Tissue samples were collected after euthanasia by using tricaine methanesulfonate (MS−222, 300 mg/L, Sigma-Aldrich). A total of 82 individuals from five wild and one cultured population of large yellow croaker were collected from different locations of China (Figure 1). The five wild populations were Zhoushan (ZS; 17 individuals) population from Zhejiang province; Fuding (FD; 16 individuals), Dayu (DY; five individuals), and Xiamen (XM; 18 individuals) populations from Fujian province; and Zhanjiang (ZJ; 16 individuals) population from Guangdong province. The cultured population was Fufa (FF; 10 individuals) population from Fujian province. A total of 53 individuals from four closely related species of large yellow croaker were collected from Fujian province of China, including eight little yellow croakers (*Larimichthys polyactis*), 10 big head croakers (*Collichthys lucidus*), eight brown croakers (*Miichthys miiuy*), nine yellow drums (*Nibea albiflora*), and eight dusky roncadors (*Megalonibea fusca*). Fin clips were collected from the fish samples, and DNA was extracted by using DNeasy Blood & Tissue Kit (Qiagen, Shanghai, China). DNA concentration was quantified by using NanoDrop 2000 (Thermo Scientific), and the integrity was checked by 1.5% agarose gel electrophoresis stained with ethidium bromide. DNA samples of large yellow croaker that fulfilled the quality requirement for whole-genome sequencing (WGS) were kept for further analyses. Sequencing libraries with 250 bp insert size were constructed using NEBNext Ultra DNA library Prep Kit (New England BioLabs, United Kingdom) according to the manufacturer’s instructions. The libraries were sequenced by using the Illumina HiSeq2500 platform with a paired-end read length of 150 bp.

### Single-Nucleotide Polymorphism Identification

The adapters, low-quality sequences (below quality 3), and short sequences (length < 30 bp) in the raw reads were trimmed using Trimmomatic-0.38 (Bolger et al., 2014). After trimming, the high-quality reads were mapped to the large yellow croaker genome (Chen et al., 2019) by using BWA version 0.7.17-r1188 (Li and Durbin, 2009) with default parameters to generate sequence alignment SAM files. SNPs were identified by using GATK version 4.0.2.1 (Mckenna et al., 2010). SNP calling was performed using standard hard filtering parameters according to GATK best practice recommendations (Der-Auwera et al., 2013) separately in each population, with the following criteria: mapping quality score ≥ 20; relevant base quality score ≥ 20; SNP quality score ≥ 20; SNP position coverage ≥ 10; minor allele frequency ≥ 5%, and missing count ≤ 3. Further filtering was performed using vcftools version 0.1.15 (Danecek et al., 2011) to remove all SNPs with minor allele counts less than 2, genotype missing counts greater than 2, or number alleles more than 3.

### Single-Nucleotide Polymorphism Selection

**FIGURE 2:**
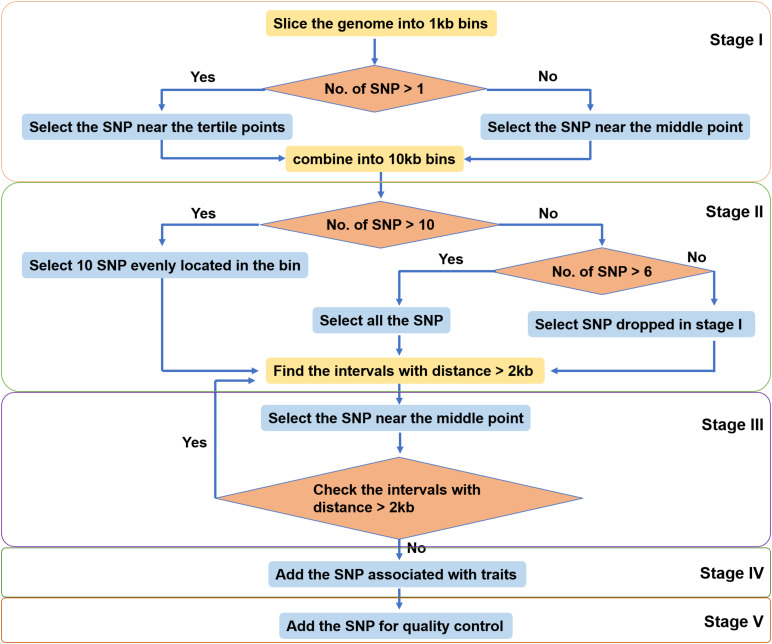
Single-nucleotide polymorphism (SNP) selection strategy in this study.

The SNPs identified in different populations of large yellow croaker were pooled together. SNPs were filtered based on the 35 bp flanking sequence with the following criteria: contain less than four contiguous G or C and less than six adjacent A or T; GC content between 30 and 70%; and contain only one SNP and no insertion or deletion. SNPs were further selected by a custom-made algorithm (Figure 2) including five stages to ensure an even distribution of SNPs over the large yellow croaker genome. In Stage I, we split the reference genome into 1-kb-sized bins. If a bin contained more than one SNP, the two SNPs most close the two drifters were selected. In Stage II, 10 adjacent bins were merged into one 10-kb-sized bin, and the number of SNPs on each new larger bin was totalized. We selected 10 SNPs uniformly in bins that contained more than nine SNPs, selected all SNPs in bins that contained more than six SNPs but less than 10 SNPs, and picked up dropped SNPs if there were less than seven SNPs in a bin. In Stage III, we scanned the whole genome to find out all gaps larger than 2 kb. Then we filled them with dropped SNPs most close to their midpoints until all gaps were narrowed within 2 kb or until there no dropped SNPs can be used. Finally, trait-related SNPs and control SNPs were added into the SNP panel. All SNPs selected to the panel were submitted to Thermo Fisher Scientific (Santa Clara, CA, United States) for array production.

### Evaluation of the Single-Nucleotide Polymorphism Array

The NingXin-I SNP array was evaluated in 96 large yellow croaker samples, and 53 fish from five closely related species in Sciaenidae including eight little yellow croakers (*L. polyactis*), 10 big head croakers (*C. lucidus*), eight brown croakers (*M. miiuy*), nine yellow drums (*N. albiflora*), and eight dusky roncadors (*M. fusca*). Genomic DNA was extracted from blood and quantified by NanoDrop 2000 (NanoDrop Technologies Inc., Wilmington, DE, United States). High-quality DNA samples were genotyped by using the NingXin-I SNP array. The genotype data were converted to Ped/Map format using a python script and then imported into PLINK software (version 1.9) (Chang et al., 2015) to conduct further analyses. A threshold with a call rate > 90% and minor allele frequency > 0.02 was set to filter out the low-quality SNPs from the raw data. A phylogenetic tree was constructed by using VCF-kit (version 0.1.6) (Cook and Andersen, 2017) with a neighbor-joining method.

## Results

### Whole-Genome Re-sequencing and Single-Nucleotide Polymorphism Calling

A total of 650 Gb raw reads were obtained from 82 large yellow croaker individuals by whole-genome re-sequencing. The average sequencing coverage for each large yellow croaker was 11.33 × . SNP identification was performed separately in each population. The SNP numbers identified in the six populations varied from 6.5 to 7.6M (Table 1). Overall, a total of 9.3M unique SNPs were identified.

**TABLE 1:** Genome re-sequencing and SNP calling for the wild and cultured population of large yellow croakers.

Sampling location	No. of individuals	Raw bases (Gb)	Average sequencing depth	No. of population- specific SNP	No. of SNP inside population
Dayu (DY)	5	71	20.28	1,686	6,506,310
Fuding (FD)	16	88	7.85	4,320	7,521,970
Xiamen (XM)	18	127	10.09	1,256	6,868,092
Zhanjiang (ZJ)	16	110	9.84	9,785	7,382,463
Zhoushan (ZS)	17	131	11.00	17,922	7,632,423
Fufa (FF)	10	123	17.58	6,869	7,549,120
Total	82	650	11.33	41,838	9,335,807

*SNP, single-nucleotide polymorphism.*

### Single-Nucleotide Polymorphism Filtering Based on Flanking Sequence and *In silico* Analysis

For quality control, SNPs were filtered based on the 35 bp flanking sequence according to the criteria described in “Materials and Methods” section. A total of 1.97M SNPs were retained after filtration. These SNPs were submitted to Affymetrix Axiom myDesign GW bioinformatics pipeline (Thermo Fisher Scientific, United States) for *in silico* analysis to predict the reproducibility. Based on the score of *in silico* analysis, the SNPs were classified into recommended, neutral, and not-recommended categories, with 0.21, 1.67, and 0.97 M SNPs, respectively. The SNPs in recommended and neutral groups were kept for further analyses.

### Single-Nucleotide Polymorphism Selection for the Final Array

**FIGURE 3:**
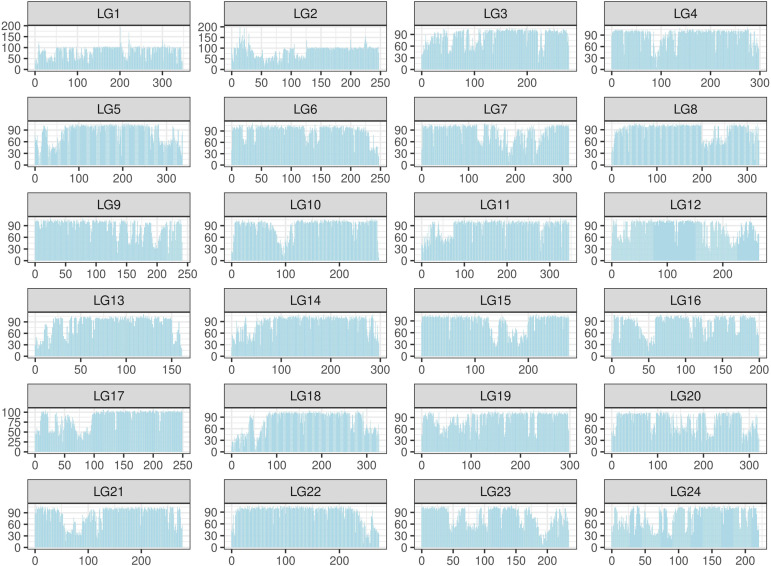
Distribution of single-nucleotide polymorphism (SNP) in the large yellow croaker genome. The distribution was calculated in 24 linkage groups by a unit (bin) of 100K base pairs. The *X*-axis represents the location in each linkage group; the *Y*-axis represents the number of SNPs.

The SNPs were filtered by a custom-made algorithm (Figure 2) to keep them evenly distributed across the large yellow croaker genome. Finally, about 579.4K SNPs were submitted to Affymetrix (Thermo Fisher Scientific, United States) for array production. SNP density was similar in the 24 chromosomes of the large yellow croaker genome (Supplementary Figure S1). The average distance between the SNPs in the large yellow croaker genome was 1.19 kb, and the vast majority of the gaps was smaller than 2K. After an analysis of the distribution of SNP by slicing the large yellow genome into 100K bins, only 60 bins contain less than 10 SNPs, accounting for 0.897% of the genome; and only four bins do not have any SNP, accounting for 0.06% of the genome (Figure 3). The classification of SNPs in the final array according to conversion type is listed in Supplementary Tables S1, S2. The recommended SNP classes (90.79% in total) including “poly high resolution,” “no minor homozygote,” “mono high resolution,” and “other” types were proceeded for further analyses according to the Axion genotyping solution data analysis guide.

### Evaluation of the NingXin-I Single-Nucleotide Polymorphism Array in Large Yellow Croaker Populations

**FIGURE 4:**
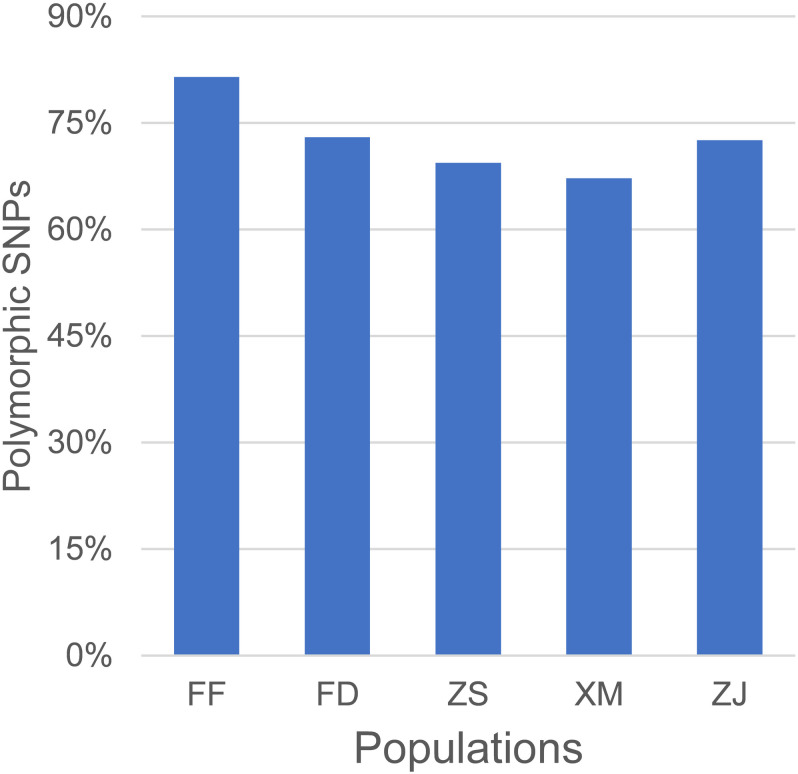
Evaluation of single-nucleotide polymorphism (SNP) array by polymorphic rate of large yellow croaker from different populations. The *X*-axis represents different populations of large yellow croakers. FD, Fuding; DY, Dayu; XM, Xiamen; ZJ, Zhanjiang; ZS, Zhoushan.

The SNP array was evaluated in different large yellow croaker populations, including 18 large yellow croakers used in the previous genome re-sequencing and 78 large yellow croakers from other populations. The percentage of 81.49, 73.00, 69.40, 67.23, and 72.60% polymorphic SNPs was identified independently from Fufa, Fuding, Zhoushan, Xiamen, and Zhanjiang populations, respectively (Figure 4). The number of population-specific SNPs varied from 1,686 to 17,922 (Table 1). A total of 483,148 (83.38%) polymorphic SNPs were identified among the five populations. The highest number of SNPs (53.24%) was located in the intergenic region, followed by the intron (39.74%) and exon (7.02%). The number of SNPs per KB was similar independent of their genome component location, ranging from 0.677 to 0.769 (Table 2).

**TABLE 2:** Distribution of available SNPs in large yellow croaker genome.

Category	Number	Percent	Total length (Mb)	Density (no. per kb)
Exon	33,179	7.02	43.25	0.767
Intron	187,807	39.74	253.78	0.740
Intergenic region	251,560	53.24	371.65	0.677
All	472,546	100.00	668.67	0.707

*SNP, single-nucleotide polymorphism.*

We also compared the genotypes generated from the whole-genome re-sequencing and SNP array. Only 0.35M genotypes (3.86%) that were genotyped using WGS were missing from the SNP array, while 1.25M (13.75%) genotypes were recovered by the SNP array, showing an overall better success rate of the SNP array than WGS on these 579K SNPs. These results suggested the genotyping accuracy of the NingXin-I SNP array in different large yellow croaker populations.

### Assessment of the NingXin-I Single-Nucleotide Polymorphism Array in Closely Related Species in Sciaenidae

**FIGURE 5:**
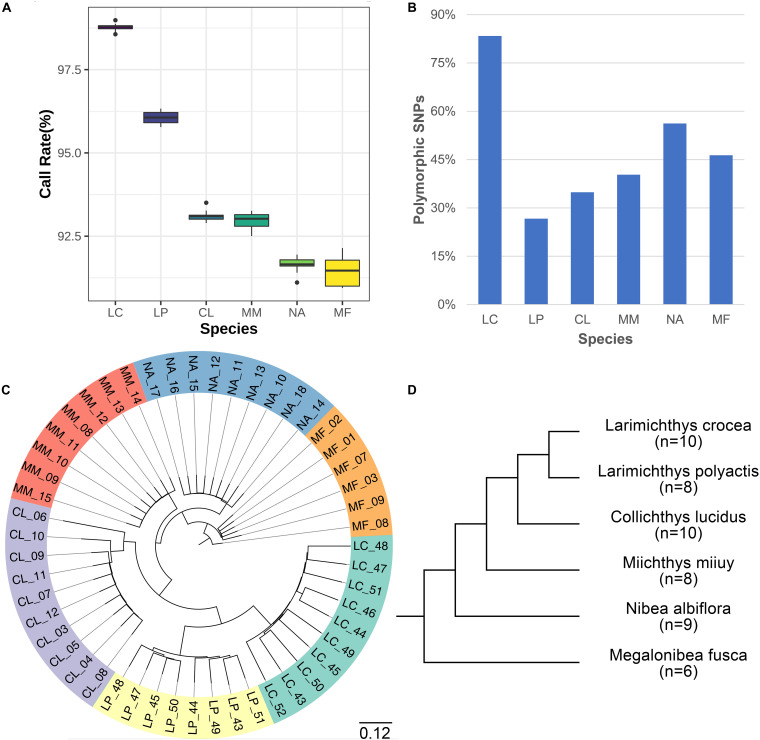
Evaluation of single-nucleotide polymorphism (SNP) array in five species in Sciaenidae that are closely related to large yellow croaker. (A) Call rate of SNPs in Sciaenidae species. **(B)** Percent of polymorphic SNPs in Sciaenidae species. **(C)** Phylogenetic tree of the species in Sciaenidae generated by using the genotyping data. **(D)** Evolutionary distance of the species in Sciaenidae by phylogenetic analysis (Modified from Jiang et al., 2014). LC, *Larimichthys crocea*; LP, *Larimichthys polyactis*; CL, *Collichthys lucidus*; MM, *Miichthys miiuy*; NA, *Nibea albiflora*; MF, *Megalonibea fusca*.

The NingXin-I SNP array was assessed in five species closely related to large yellow croaker in Sciaenidae including eight little yellow croaker (*Larimichthys polyactis*), 10 big head croaker (*Collichthys lucidus*), eight brown croaker (*Miichthys miiuy*), nine yellow drum (*Nalbiflora albiflora*), and eight dusky roncador (*Megalonibea fusca*). The percentage of working SNPs (call rate > 0.9) was 95.86, 95.01, 89.32, 88.82, 87.72, and 87.45% in large yellow croaker, little yellow croaker, big head croaker, brown croaker, yellow drum, and dusky roncador, respectively (Figure 5A). The number of polymorphic SNPs ranged from 154.6K to 483.1K (Figure 5B), and the species-specific SNPs varied from 3.71K to 103.59K in the six species in Sciaenidae (Supplementary Table S3). Population structure of Sciaenidae species using principal component analysis (PCA) formed six clusters (Supplementary Figure S2), which indicated that these species were apart from each other. A phylogenetic tree generated by using the genotype data of the NingXin-I SNP array indicated that little yellow croaker was the closest species with large yellow croaker, followed by the big head croaker, brown croaker, yellow drum, and dusky roncador (Figure 5C), which was consistent with the phylogenetic distances calculated by using mitochondrial gene CO1 (Figure 5D; Jiang et al., 2014).

## Discussion

SNP array technologies represent an efficient and robust tool for genome-scale genotyping, which have been developed in many fish species, significantly enhancing aquaculture genetics and breeding research. In this study, we developed and evaluated the first high-throughput SNP array NingXin-I for large yellow croaker. The NingXin-I SNP array is a powerful genetic tool for GWAS and to analyze the genetic architecture of different traits of large yellow croaker.

A comprehensive source of candidate SNPs is the foundation of high-quality SNP array development. About 139 million putative SNPs were detected by re-sequencing 243 chickens from 24 chicken lines during the development of a high-density 600K SNP genotyping array for chicken (Kranis et al., 2013). Recently, around 7.09–9.41 million SNPs were detected in eight representative local breeds or commercial broiler lines in the development of a new chicken 55K SNP genotyping array (Liu et al., 2019). Nearly 12 million candidate SNPs were discovered during the development of the SNP array for Pacific and European oysters (*Crassostrea gigas* and *Ostrea edulis*) (Gutierrez et al., 2017). About 24.2 million potential SNPs were identified in common carp (Xu et al., 2014). A total of over 9.6 million putative SNPs were identified during the development of the 690K SNP array in catfish (Zeng et al., 2017). SNP numbers in each species may vary by genome size, complexity, chromosomes ploidy, and the number of families or strains. The 9.3 million non-redundant SNPs identified in large yellow croakers were sufficient for the development of our 600K SNP array. In order to get a comprehensive source of SNPs, 82 individuals from five wild and one cultured population of large yellow croaker were collected and re-sequenced from six different locations of China. These locations covered the three putative geographical stocks, i.e., Daiquyang, Min-Yuedong, and Naozhou stocks along the coastal waters of China (Liu and De Mitcheson, 2008). A similar call rate was observed in the wild stocks and cultured population during the subsequent evaluation of the SNP array, indicating the stability and accuracy of the NingXin-I SNP array across different large yellow croaker populations. The large yellow croaker samples collected from wild stocks and cultured population, combined with the *de novo* whole-genome re-sequencing technique, guaranteed that the developed NingXin-I SNP array will perform well across different populations and that it will be broadly useful for population genetics and genome-assisted breeding of large yellow croaker. Medium- to high-density SNP arrays ranging from 1.5K to 690K have been developed in aquaculture species (Zenger et al., 2019). With 600K SNPs, this array is in the upper end of aquaculture SNP arrays, showing significant lower genotyping costs per SNP than low- and medium-density arrays. A high polymorphic SNP rate (83.38%) was observed during the evaluation of NingXin-I in the wild and cultured large yellow croaker populations. Compared with the other aquaculture species, about 67.5% polymorphic SNPs were identified during the assessment of the 690K catfish array (Zeng et al., 2017); 64.5 and 86.0% polymorphic SNPs were identified in the 50K (Salem et al., 2018) and 57K (Palti et al., 2015) rainbow trout arrays, respectively, 74.0 and 83.4% polymorphic SNPs were identified in the 58K (Joshi et al., 2018) and 65K (Peñaloza et al., 2020) Nile tilapia arrays, respectively; 70.4% polymorphic SNPs were identified in the 190K Pacific oyster array (Qi et al., 2017); 74.6% polymorphic SNPs were identified in the 38K combined-species SNPs array for Pacific and European oysters (Gutierrez et al., 2017); 67.5% polymorphic SNPs were identified in the 9K Pacific white shrimp array (Jones et al., 2017); 70.6% polymorphic SNPs were identified in the 6K black tiger shrimp array (Baranski et al., 2014); 79.6% polymorphic SNPs were identified in the 200K Atlantic salmon array (Yáñez et al., 2016); 74.06% polymorphic SNPs were identified in the 250K common carp array (Xu et al., 2014); and 74.7% polymorphic SNPs were identified in the 50K Japanese flounder array (Zhou et al., 2020). The polymorphic SNP rate of NingXin-I (83.38%) is one of the highest among aquaculture SNP arrays, although others have achieved also a high polymorphism rate, such as rainbow trout with 86.0%, and Nile tilapia with 83.4%. Although the SNP genotyping rate and polymorphic SNP rate may change with software parameters, population diversity, and sample size, the high polymorphic SNP rate across wild and cultured populations of large yellow croaker compared with other species still provides solid evidence of the good design and reliability of the NingXin-I array. A comparison of the genotypes collected from the whole-genome re-sequencing and NingXin-I SNP array was conducted. About 13.75% genotypes were recovered by the SNP array, which was absent in WGS; only 3.86% alleles genotyped using WGS were missing in the SNP array, which proved an overall better success rate of the SNP array than whole-genome re-sequencing in this study. High consistency of the NingXin-I SNP array with the WGS data provides strong support for highly reliable genotypic data across all validated SNPs. Although increasing the sequencing depth of WGS may improve the allele and SNP genotyping rate and accuracy, the cost would be increased significantly. For population scale genotyping studies, the NingXin-I SNP array would be more cost-efficient and accurate than WGS. A large proportion (>87.45%) of the SNPs worked in large yellow croaker, little yellow croaker, big head croaker, brown croaker, yellow drum, and dusky roncador during the assessment of the NingXin-I SNP array. The SNP genotyping rate was higher in species with closer phylogenetic distance with large yellow croaker. However, no similar pattern was observed between the polymorphic SNP rate and phylogenetic distance among the six species, which may have been caused by the relatively small sample size of the other Sciaenidae species. The small size of samples for little yellow croaker, big head croaker, brown croaker, yellow drum, and dusky roncador was genotyped in this study, because of significantly decreased wild population size of these species. So the number of polymorphic and species-specific SNPs may change with different populations and number of tested animals in Sciaenidae species in the future studies. The highly similar phylogenetic trees generated by using the genotype data of the NingXin-I array, and the mitochondrial gene CO1 (Jiang et al., 2014), and the species taxonomy by using morphological data (Taniguchi, 1969a, b; Taniguchi, 1970) proved the genotyping accuracy of the NingXin-I SNP array in Sciaenidae. The successful genotyping of closely related species in Sciaenidae suggested that the NingXin-I SNP array could be potentially used in these species, demonstrating the broad application of the NingXin-I SNP array.

Genotype imputation from very low to medium density has been proved to be a cost-effective tool for GS in Atlantic salmon breeding programs, and reducing SNP density had little impact on prediction accuracy until 5,000 SNPs (Tsairidou et al., 2020). In the near future, a second-generation SNP array named NingXin-II with 50K SNPs for large yellow breeding will be developed. The NingXin-II will be developed based on NingXin-I with lower throughput to reduce the array manufacturing cost. The NingXin-II array will integrate the representative high-quality polymorphic SNP in each haplotype block and SNPs associated with multiple traits. The NingXin-II array with low manufacturing cost and high specificity and accuracy will be used in the genome-based breeding of large yellow croaker.

## Conclusion

The first high-throughput genotyping SNP array NingXin-I for large yellow croaker was developed. After an evaluation with numerous samples of large yellow croakers from multiple populations, 83.38% of the SNPs were polymorphic sites. Besides, the array was also assessed in other closely related Sciaenidae species, and the polymorphic SNP rate ranged from 26.68 to 56.23%. These results suggested the stability, accuracy, and potential broad application of the NingXin-I array. This study indicates that the NingXin-I SNP array will be valuable for genetic studies and breeding in large yellow croaker and related species.

## Data Availability Statement

The sequencing data is available at the NCBI under accession number: PRJNA629649. The genotype of large yellow croaker and code for SNP selection are available at FigShare (https://doi.org/10.6084/m9.figshare.12937079).

## Ethics Statement

The animal study was reviewed and approved by the Laboratory Animal Management and Ethics Committee, College of Ocean and Earth Sciences, Xiamen University. All experimental procedures involving large yellow croaker and the other fishes were performed according to the Regulations for the Administration of Affairs Concerning Experimental Animals (College of Ocean and Earth Sciences, Xiamen University).

## Author Contributions

PX and WZ conceived and supervised the study. TZ, BC, and PX designed, managed the experiments, and wrote the manuscript. QK, JZ, FP, YW, and LC conducted the experiment. ZZ, YB, YP, and JG performed the analysis and designed the charts and tables. All authors have read and approved the manuscript.

## Conflict of Interest

QK, YP, and WZ were employed by the Ningde Fufa Fisheries Company Limited. The remaining authors declare that the research was conducted in the absence of any commercial or financial relationships that could be construed as a potential conflict of interest.

## Supplementary Material

Supplementary Figure 1 Density of SNP in the large yellow croaker genome. The *X*-axis represents the location of the linkage group; the *Y*-axis represents the number of SNP.

Supplementary Figure 2 Population structure of Sciaenidae species using PCA analysis. large yellow croaker *Larimichthys crocea* LC, little yellow croaker *Larimichthys polyactis* LP, big head croaker *Collichthys lucidus* CL, brown croaker *Miichthys miiuy* MM, yellow drum *Nibea albiflora* NA, dusky roncador *Megalonibea fusca* MF.

Supplementary Table 1 Classification of SNPs in the final array according to conversion type.

Supplementary Table 2 The position and conversion type of SNPs in the Ningxin-I array.

Supplementary Table 3 Assessment of the Ningxin-I SNP array in closely related species in Sciaenidae.

## Abbreviations

GBS: genotyping by sequencing
GS: genomic selection
GWAS: genome-wide association study
MAC: minor allele count
MAF: minor allele frequency
MAS: marker- assisted selection
QTL: quantitative trait loci
RAD Seq: restriction site-associated DNA sequencing
SNP: single-nucleotide polymorphism.
