# Supplementary figures and images for: Development and Evaluation of a High-Throughput Single-Nucleotide Polymorphism Array for Large Yellow Croaker (Larimichthys crocea)
Source: Front Genet. 2020 Oct 23;11:571751. doi: 10.3389/fgene.2020.571751 (PMC7645154; doi:10.3389/fgene.2020.571751)

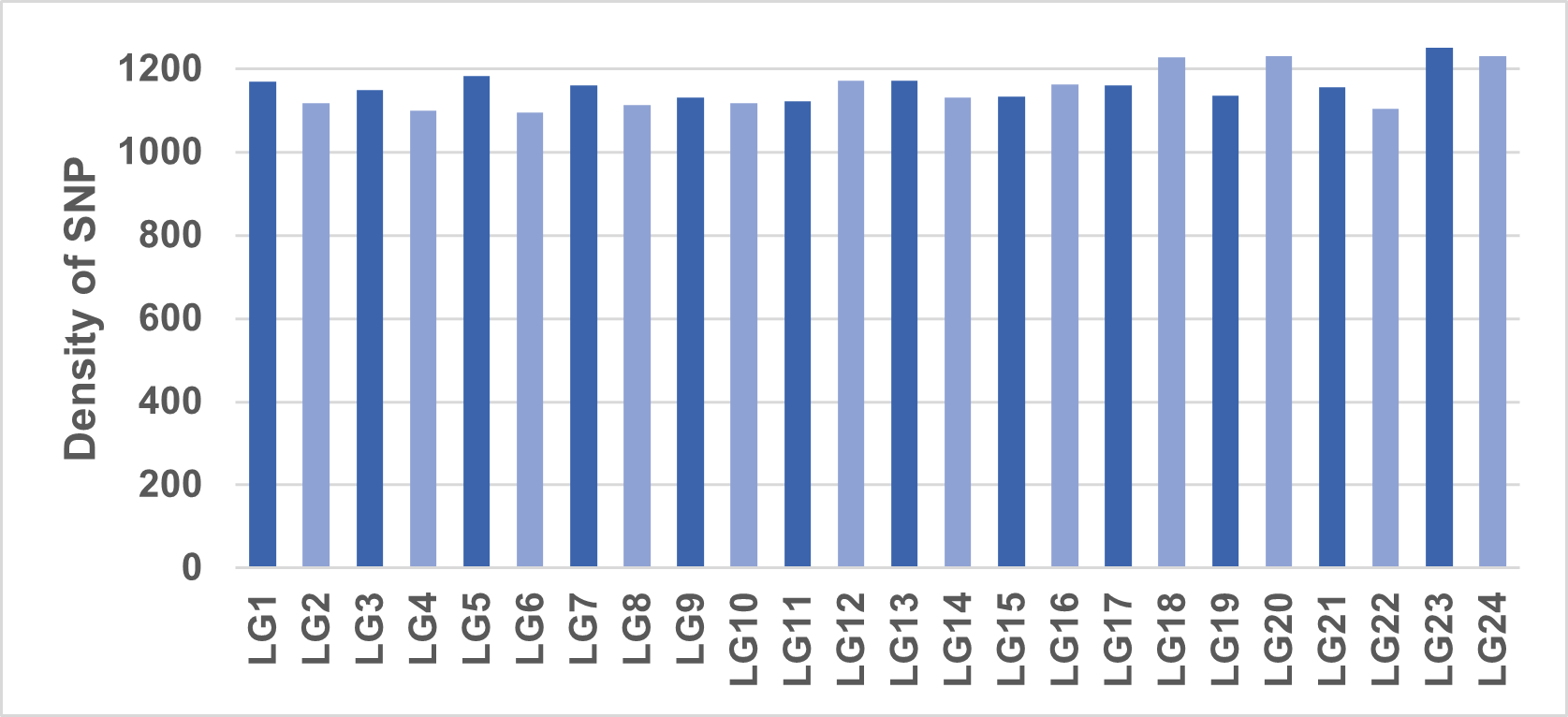

Supplement: Supplementary Figure 1 — Density of SNP in the large yellow croaker genome. The X-axis represents the location of the linkage group; the Y-axis represents the number of SNP. [file Image_1.TIF]

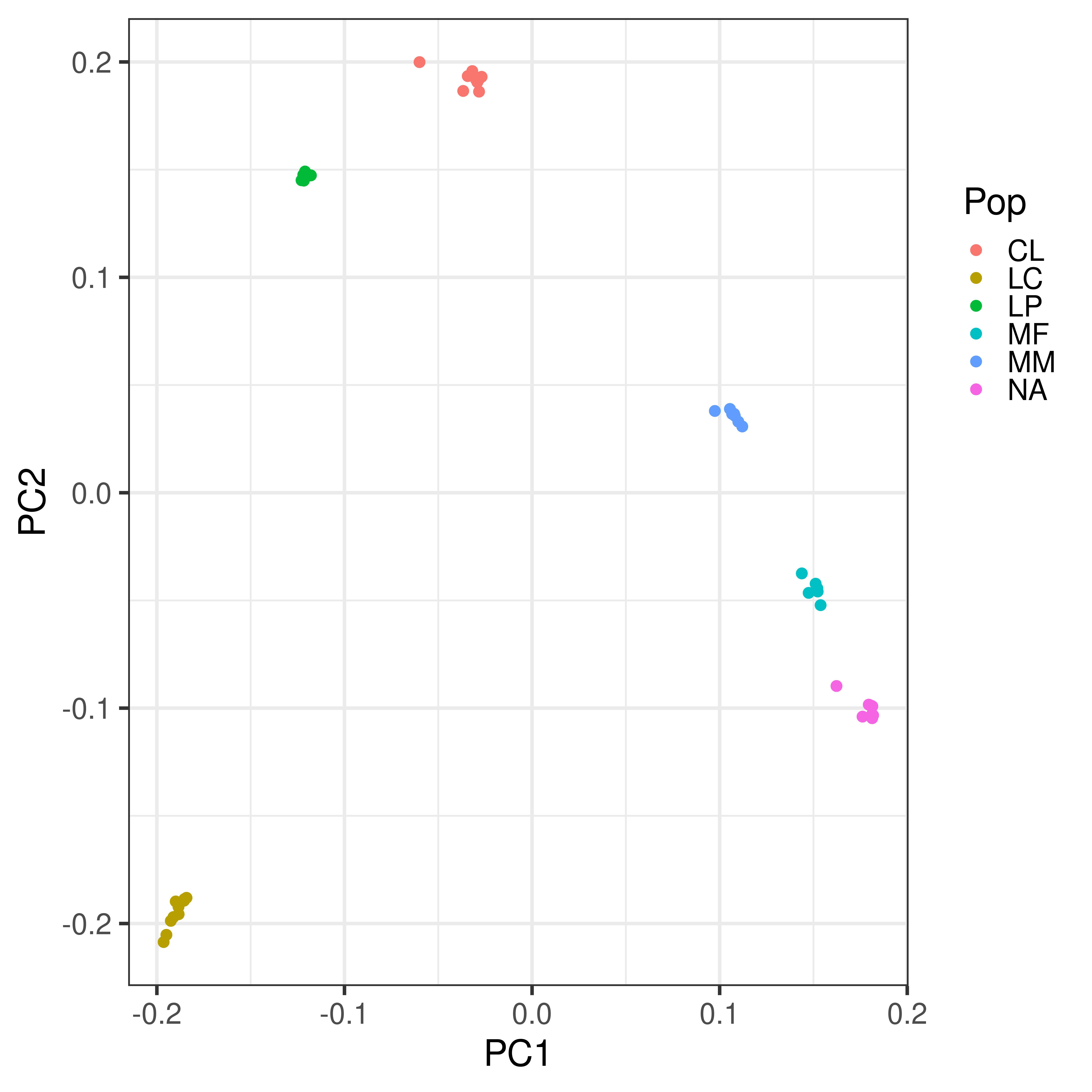

Supplement: Supplementary Figure 2 — Population structure of Sciaenidae species using PCA analysis. large yellow croaker Larimichthys crocea LC, little yellow croaker Larimichthys polyactis LP, big head croaker Collichthys lucidus CL, brown croaker Miichthys miiuy MM, yellow drum Nibea albiflora NA, dusky roncador Megalonibea fusca MF. [file Image_2.JPEG]
