# Supplementary material for: Development and Evaluation of a High-Throughput Single-Nucleotide Polymorphism Array for Large Yellow Croaker (Larimichthys crocea)
Source: Front Genet. 2020 Oct 23;11:571751. doi: 10.3389/fgene.2020.571751 (PMC7645154; doi:10.3389/fgene.2020.571751)
Supplement: Supplementary Table 1 — Classification of SNPs in the final array according to conversion type. [file Table_1.DOCX]

**Table S1 |** Classification of SNPs in the final array according to conversion type.

| Conversion Type | Count | Percentage |
| --- | --- | --- |
| Poly high resolution | 335,586 | 57.91% |
| No minor homozygote | 111,105 | 19.17% |
| Other | 62,626 | 10.81% |
| Mono high resolution | 16,757 | 2.89% |
| Call rate below threshold | 35,450 | 6.12% |
| Off target variant | 17,948 | 3.10% |
| Total | 579,472 | 100.00% |
