# Supplementary material for: Development and Evaluation of a High-Throughput Single-Nucleotide Polymorphism Array for Large Yellow Croaker (Larimichthys crocea)
Source: Front Genet. 2020 Oct 23;11:571751. doi: 10.3389/fgene.2020.571751 (PMC7645154; doi:10.3389/fgene.2020.571751)
Supplement: Supplementary Table 2 — The position and conversion type of SNPs in the Ningxin-I array. [file Table_2.DOCX]

**Table S2** **|** Assessment of the Ningxin-I SNP array in closely related species in Sciaenidae.

| Species | Common name | No. of species-specific SNPs | No. of polymorphic SNPs | Call rate |
| --- | --- | --- | --- | --- |
| *Larimichthys crocea* | large yellow croaker | 103,592 | 483,148 | 95.86% |
| *Larimichthys polyactis* | little yellow croaker | 4262 | 154,580 | 95.01% |
| *Collichthys lucidus* | big head croaker | 4829 | 202,113 | 89.32% |
| *Miichthys miiuy* | brown croaker | 3713 | 233,663 | 88.82% |
| *Nibea albiflora* | yellow drum | 7656 | 325,831 | 87.72% |
| *Megalonibea fusca* | dusky roncador | 6478 | 268,649 | 87.45% |
